# Using benzodiazepines and Z-drugs for managing primary insomnia in adults in Saudi Arabia: an e-Delphi study to aid the development of clinical guidelines

**DOI:** 10.1007/s11325-019-01794-7

**Published:** 2019-02-07

**Authors:** Ali Dobia, Kath Ryan, Ahmed S. BaHammam, Alexander Edwards

**Affiliations:** 1 Reading School of Pharmacy, University of Reading, Whiteknights, 226, Reading, RG6 6AP UK; 2 General Directorate of Medical Services, Ministry of Interior, Olaya, Riyadh, Saudi Arabia; 3 University Sleep Disorders Center, College of Medicine, King Saud University, Riyadh, Saudi Arabia; 4 Reading School of Pharmacy, University of Reading, Whiteknights, 224, Reading, RG6 6AD UK

**Keywords:** Benzodiazepines, Z-drugs, Saudi Arabia, Clinical guidelines, Primary insomnia, e-Delphi technique

## Abstract

**Purpose:**

This study aims to obtain consensus statements required for the development of clinical guidelines for the use of benzodiazepines (BZDs) and Z-drugs for the management of primary insomnia in adults in Saudi Arabia.

**Methods:**

Three rounds of the e-Delphi technique using a Bristol Online Survey (BOS) were conducted between May and August 2018. The Director of the Saudi Sleep Medicine Group helped recruit the country’s sleep medicine experts. Snowballing was used to forward invitation emails, information sheets, and the survey to known sleep medicine experts and physicians deemed to be interested in the field. All participants’ details were anonymised except to the researcher.

**Results:**

Fifteen experts from four different regions and specialities in Saudi Arabia participated in Round 1. Twenty-one statements originated from participants’ responses. In Round 2, there were 17 respondents and 16 of the statements obtained the required consensus of 70% or higher. Eleven experts participated in Round 3 and eight statements received 100% agreement, two received 91%, and six received 82%. Having obtained the required consensus of 80% or higher in Round 3, these 16 statements fulfilled the criteria to be included in future guidelines. The five statements that failed to attain the required consensus were rejected as inappropriate for inclusion in Saudi Arabian clinical guidelines.

**Conclusions:**

The items that achieved the required consensus can be included in future guidelines for the use of BZDs and Z-drugs in the treatment of primary insomnia in adults to standardize best practices in sleep medicine in Saudi Arabia.

## Introduction

Insomnia and other sleep disorders are amongst the most neglected illnesses by medical practitioners [1]. The various studies that have attempted to address the prevalence of sleep disorders in the Kingdom of Saudi Arabia are limited [2]. Nevertheless, according to the available data, it is evident that sleep disorders are increasingly becoming prevalent amongst Saudis, yet sleep medicine services in Saudi Arabia remain below the level of services offered in developed countries [3]. A recent study reported the prevalence of insomnia with the presence of daytime dysfunction in 57% of Saudi adults attending primary care services [1]. Various obstacles have been cited as the factors that hinder the progress of speciality in sleep medicine in Saudi Arabia. They include a lack of specialists, few trained technicians, and insufficient funding [2]. Furthermore, awareness about insomnia and other sleep disorders as well as their consequences is low amongst healthcare authorities and practitioners [1]. This lack of knowledge is attributed to the poor education received by medical students who transition into practice [4]. The low awareness is also widespread amongst the general public, health care workers and authorities, and the insurance companies in Saudi Arabia [2]. With such low awareness and knowledge about insomnia, there are no evidence-based clinical guidelines regarding the management of the illness amongst Saudis.

Overall, two treatment options have been accepted widely for the management of insomnia. These methods include cognitive behavioural therapy for insomnia (CBT-I) and hypnotic medications [5, 6]. Comparatively, hypnotic drugs act faster than CBT-I and are thus preferred by patients [6]. Some of the drugs used in the treatment of insomnia are benzodiazepines and Z-drugs. Despite being indicated for this disorder, benzodiazepines have the potential to trigger dependence with consequent rebound and withdrawal symptoms upon sudden discontinuation [6]. In contrast to benzodiazepines, Z-drugs are more selective in their action and have a lower tendency to develop dependence and withdrawal symptoms [6]. They still, however, have similar adverse effects associated with benzodiazepines as they can cause anterograde amnesia, sedation, impaired balance, and complex sleep-related behaviour [6, 7].

Hypnotic risks and side effects have led to the development of many guidelines for diagnosing and managing chronic insomnia in different countries [8–12]. Whilst benzodiazepines and Z-drugs have proven benefits in the management of primary insomnia, contradiction and differing opinions surround their use [13]. This is because there is no clear evidence guiding the judicious use of these drug classes for the treatment of insomnia, especially in countries like Saudi Arabia where there is an overall low awareness of sleep medicine [2]. In addition, many physicians in Saudi Arabia do not follow international guidelines because they lack awareness of them or think they are culturally inappropriate for use in Saudi Arabia [14]. It is, therefore, imperative that Saudi Arabia has its own guidelines for treating and managing insomnia and other sleep disorders, which are consistent with its own history and culture.

Consensus methods can be used to come up with guiding principles to eliminate the barriers associated with contradictory opinions [15]. One such approach is the Delphi technique, a structured process designed to arrive at a consensus of choice or judgement following responses by a panel of experts to rounds of questionnaires on a topic for which there is little evidence [16, 17]. This method is particularly useful in situations where several dissenting opinions and contradictions surround a subject [15, 16], as in the use of Z-drugs and benzodiazepines in the management of primary insomnia in Saudi Arabia. Guidelines developed by this means are likely to be accepted widely by medical practitioners in the country involved [15].

## Methods

The purpose of this study was to use the e-Delphi method to create a set of statements that could be used to develop a clinical guideline for the use of Z-drugs and benzodiazepines for managing primary insomnia (difficulty to sleep without a known cause) [11] amongst adults in Saudi Arabia.

### Delphi technique

The consensus process incorporated three rounds e-Delphi technique [16], using Bristol online survey, which took place between May and August 2018. Ethical approval (number 17/15) was obtained from University of Reading Ethics Committee (UREC), United Kingdom. Experts in sleep medicine in Saudi Arabia, defined as those who are specialists in sleep medicine (accredited by the Saudi Commission for Health Specialties) or work in sleep clinics or have experience of treating patients with insomnia, were recruited with the help of the Saudi Sleep Medicine Group. Snowballing was also used as participants were asked to forward an invitation email including an information sheet and a link to the survey to other physicians and/or experts in sleep disorders when they thought might be interested in participating. Participation in the first-round questionnaire was considered as consent to participate in the study. All participants’ details were anonymised, except to the first author.

#### Round 1

The first round online questionnaire included open-ended questions to solicit some information from participants [18] about their current practices and thoughts for managing primary insomnia, which might be appropriate for inclusion in consensus statements. The objective of the study and some specific instructions were provided for participants. Some demographic details were asked as well. The survey was piloted with five people (three pharmacists and two physicians) not participating in the work or in the main e-Delphi study. The purpose of the pilot was to ensure feasibility of the procedure, clarity of the questions, and to estimate the time for completing the questionnaire. No amendments were requested in Round 1. Potential participants were sent the questionnaire by email and the survey was accessible for 1 month. Responses from each question of the first round were aggregated, and then frequencies and percentages were calculated when appropriate to identify the most frequent answers. All data were tabulated together with qualitative responses as feedback for participants in the next round.

#### Round 2

Before completing the second questionnaire, each participant reflected on his/her answers compared to those of other participants. The researcher refined all responses from Round 1 and created items for ranking based on the participants’ answers and best-practice guidelines for managing primary insomnia in adult patients. Twenty-one statements were prepared for circulation in Round 2. A second online questionnaire was sent to participants, who were asked to forward it to other experts regardless of whether they had participated in Round 1. Participants were asked to rank each item on a 1 to 5 Likert scale (definitely disagree to definitely agree) that best represented their opinions about the criteria that they wanted to be included in future Saudi Arabia guidelines. They were also asked to briefly comment on their reasons for choosing a specific grade (1–5). The consensus was defined as 70% or higher agreement. The questionnaire was piloted with three physicians to ensure the procedure’s feasibility and the clarity of the statements. Of the 21 statements sent in the e-Delphi pilot, three were clarified according to the participants’ comments. All of those three physicians participated in Round 2, and the survey was accessible for 3 weeks.

#### Round 3

**Fig. 1:**
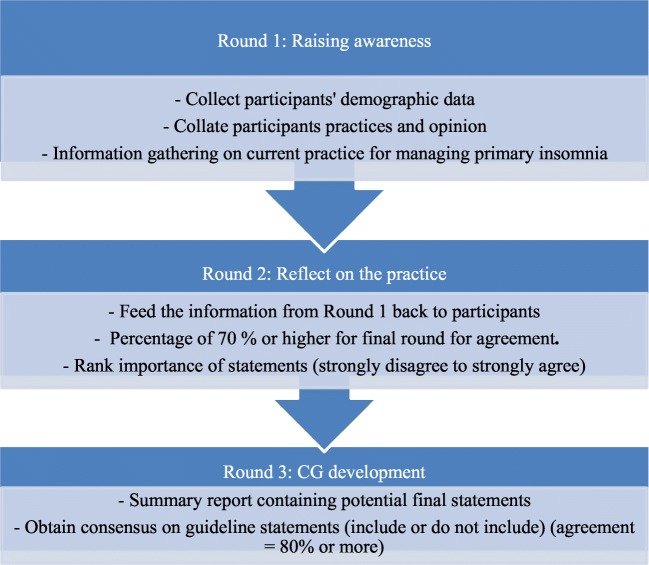
e-Delphi process to achieve consensus on statements to be included in future guidelines

Responses from Round 2 were collated and refined. Participants received feedback with ratings summarised by the researcher so that they could revise their judgements. A third questionnaire was then sent, which included 16 statements. Participants were asked to specify which of these should be included or excluded in future guidelines. Statements that were excluded because they did not achieve 70% agreement in the second round were provided for participants in a separate question, and they were asked if any statement should be reinstated and included in the guidelines. Consensus was defined as 80% or higher agreement (Fig. 1 shows e-Delphi process to achieve consensus on statements to be included in future guidelines). The survey was accessible for a month.

## Results

### Round 1

Fifteen participants from the four largest regions in Saudi Arabia (East, West, South, and Central) participated in Round 1. Eight of the participants stated that they were qualified from outside Saudi Arabia and the rest were from inside. Details of demographic data are provided in Table 1. Responses from this round lead to forming 21 statements to be ranked.

**Table 1:** Demographic data and characteristics of participants (Rounds 1, 2, and 3)

	Round 1	Round 2	Round 3
Gender	Male: 14 Female: 1	Male: 16 Female: 1	Male: 9 Female: 2
Expert in sleep medicine	Yes: 8 No: 7	Yes: 17 No: 0	Yes: 11 No: 0
Years of practice as a sleep medicine specialist or in a sleep clinic	< 5 years: 5 5–10 years: 4 > 10 years: 6	< 5 years: 8 5–10 years: 3 > 10 years: 6	< 5 years: 4 5–10 years: 3 > 10 years: 4
Region	South: 6 East: 1 West: 2 Central: 6	South: 4 East: 1 West: 1 Central: 11	South: 4 East: 1 West: 1 Central: 5
Place of practice	University hospital/sleep centre: 4 Ministry of health: 7 Private: 2 Military hospital: 2	University hospital/sleep centre: 7 Ministry of health: 6 Private: 2 King Faisal specialist hospital and research centre: 2	University hospital/sleep centre: 5 Ministry of health: 4 Private: 1 King Faisal specialist hospital and research centre: 1

### Round 2

Twenty-one statements were sent for ranking in Round 2. Seventeen responses were received and all who participated in this round were specialised or trained in sleep medicine. The data were analysed using in the BOS that showed the percentage that each statement achieved. Each statement that achieved 70% or higher from summation of score 4 and 5 on the Likert scale was considered as agreement. Sixteen statements made the cutoff for consensus agreement and five statements failed to achieve the 70% agreement in this round. Feedback from this round about the statements, each item’s rankings, and the participants’ choices were prepared and circulated to the expert panel, allowing each participant to reflect on his/her answers in relation to those of other participants before completing the third questionnaire.

### Round 3

In Round 3, 11 responses were received and all participants were experts in sleep medicine (specialised, trained in sleep medicine, or working in sleep clinics). Table 1 shows the demographic details and characteristics of the participants in each round.

All statements (16) that were returned to the expert panel in this round made the cutoff of 80% or higher consensus agreement. Table 2 shows the statements sent in Round 3 and the percentage agreements achieved. None of the excluded statements achieved the percentage agreement to be reinstated. Table 3 shows the statements that did not achieve consensus in Rounds 2 and 3. Anonymity was retained during the study.

**Table 2:** Statements achieving 80% agreement or higher, to be included in future guidelines

Statements	
100% agreement	
1. When using benzodiazepines or Z-drugs to treat primary insomnia, the diagnosis should be recorded	
2. Cognitive Behavioural Therapy for Insomnia (CBT-I) is effective and recommended for primary insomnia as first-line treatment	
3. Benzodiazepines or Z-drugs are recommended for primary insomnia for short-term use only	
4. When prescribing benzodiazepines or Z-drugs beyond the maximum treatment period, reasons for continuing should be documented	
5. When prescribed benzodiazepines or Z-drugs are ineffective, alternative medicines should be used	
6. When initiating benzodiazepines or Z-drugs for a patient, inform the patient that it will be for a limited duration	
7. When prescribing benzodiazepines or Z-drugs for long term, patients should be reviewed regularly, at least every 4–6 weeks	
8. When withdrawing patients on long-term use of benzodiazepines or Z-drugs, tapering should be considered	
91% agreement	
1. Extension beyond the maximum treatment period of benzodiazepines or Z-drugs should not take place without re-evaluating the patient	
2. Short-term hypnotic treatment should be supplemented with CBT-I when possible	
82% agreement	
1. Sleep hygiene is effective and recommended in the treatment of primary insomnia as first-line treatment	
2. Benzodiazepines and Z-drugs should be used to treat primary insomnia only when it is severe, disabling, or causing extreme distress	
3. Benzodiazepines or Z-drugs should be prescribed in the first instance with the lowest effective dosage used	
4. Benzodiazepines or Z-drugs should not be prescribed for more than 4 weeks	
5. Benzodiazepines or Z-drugs should not be prescribed for patients with a history of addiction or substance abuse	
6. Switching from one hypnotic to another should occur only if a patient experiences adverse effects directly related to a specific agent

**Table 3:** Statements eliminated from inclusion in future guidelines

Statements	Percentage achieved
Short-acting benzodiazepines or Z-drugs are recommended as the first-line pharmacological treatment for primary insomnia	25
Only Z-drugs (e.g. zolpidem) are recommended as the first-line pharmacological treatment for primary insomnia	0
When prescribed benzodiazepines or Z-drugs are ineffective, the dose should not be increased	12.5
When prescribed benzodiazepines or Z-drugs are ineffective, the dose should be increased	25
When prescribed benzodiazepines or Z-drugs are ineffective, a combination with other sedative agents can be used	37

## Discussion

Clinical practice guidelines form critical frameworks for the summary and translation of continually changing evidence from research into actual practice [19]. They assist practitioners in making reasonable clinical decisions about appropriate healthcare for specific clinical circumstances [20, 21]. This study aimed to obtain consensus on items required for developing clinical guidelines for using benzodiazepines and Z-drugs for managing insomnia amongst adults in Saudi Arabia. After three rounds of review, the e-Delphi technique generated 16 statements to be included in future guidelines. Five statements failed to reach the 80% level of consensus needed and were, therefore, excluded from the guidelines. The e-Delphi technique was considered the most appropriate method considering the lack of national clinical guidelines currently in the Saudi healthcare literature. This method facilitated the consolidation of information from experts in several locations and omitted the burden of travelling around the country [15, 16]. Because the researcher could provide feedback after each round, encourage participants to reflect on their answers, and assess them compared to other answers, the e-Delphi technique enhanced an interactive exchange of information between participants [16]. The expert panel agreed that the use of benzodiazepines and/or Z-drugs for primary insomnia should be accompanied by a documented diagnosis. The panel also agreed that before initiating any intervention, it is imperative to conduct a thorough assessment of the patient to identify all the possible causes of disturbed sleep and to identify all the salient exacerbating factors so that appropriate treatment is indicated as necessary [22]. The panel also came to the consensus that CBT-I is useful and recommended as a first-line treatment for primary insomnia. The management of primary insomnia amongst the adult population in Saudi Arabia should centre, at least initially, on cognitive and behavioural non-pharmacological approaches. These strategies include sleep hygiene, straightforward advice, relaxation techniques, counselling, and behavioural therapy [8]. Behavioural and psychological interventions have been proven to be useful for all adults as well as for chronic users of hypnotic drugs [8]. When the initial behavioural or psychological treatment proves ineffective, other approaches should be considered and the patient evaluated for potential occult comorbidities [11].

Thus, the expert consensus is that pharmacological interventions should be considered when non-pharmacological treatments were unsuccessful and alternated from one class to the other only if they prove unsuccessful. Importantly, the choice of benzodiazepines or Z-drugs should be guided by the treatment goal, symptomatology, patient preference, past response patterns, cost, comorbidities, interactions with concomitantly administered medications, side effects, contraindications, and the availability of other treatment options [11]. Although short- to intermediate-acting benzodiazepines or Z-drugs are recommended for adults with primary insomnia [6, 11], all of the expert opinions reviewed by the study emphasised that these drugs should be used for short-term use only.

Due to dependency and tolerance associated with most hypnotic medications, the panel recommended a maximum treatment duration of 2 weeks. Repeat or additional prescriptions for benzodiazepines or Z-drugs should be avoided because their long-term use can complicate prognoses [22]. Furthermore, it was agreed that dosage tapering should be considered for patients withdrawing from these drugs and that CBT-I is known to be effective in helping medication tapering and discontinuation [11].

The e-Delphi technique further revealed that all the experts involved were of the opinion that when prescribing benzodiazepines and Z-drugs beyond the maximum treatment period, reasons for continuing should be documented. Long-term hypnotic therapy may be indicated for individuals with refractory or severe insomnia or persons with chronic comorbidities [11]. Consistent follow-up, however, is imperative, preferably every 4–6 weeks in the initial phase of treatment, to assess potential side effects, treatment efficacy, and the reason for continuing the medication [11]. The prescriber should always ensure that hypnotic medications are reviewed regularly and all review dates and relevant advice are clearly documented in the patient’s records [22].

The panel was unanimous that alternative medicines should be used if the prescribed benzodiazepines or Z-drugs are deemed to be ineffective. Drugs from other classes are available to treat primary insomnia, but unlike benzodiazepines and Z-drugs, they work through receptors other than the benzodiazepine section of the gamma-aminobutyric acid (GABA-A) receptor [6]. Additionally, the experts agreed that patients should be informed that using benzodiazepines or Z-drugs is for a limited duration only. Patient education should always accompany treatment, particularly if benzodiazepines and Z-drugs are prescribed. Amongst the many factors that patients should be informed about are safety concerns, treatment expectations and goals, potential drug interactions and side effects, alternative treatment modalities including behavioural and cognitive therapy, rebound insomnia, and the possibility for dosage increments [11].

Over 90% of the responses given recommended that extension beyond the maximum treatment period for benzodiazepines or Z-drugs should not take place without re-evaluation of the patient and that short-term hypnotic treatment should be supplemented with CBT-I when possible. Furthermore, patients should be advised to keep sleep diaries and that, should a relapse occur, the data be used for long-term re-evaluation [11]. In addition to clinical reassessment of patients, regular administration of survey tools, such as questionnaires, might be useful in outcome assessment. Findings from such tools would help in informing subsequent treatment efforts [11].

Most experts consider sleep hygiene a useful treatment modality and recommend it as a first-line treatment for insomnia. Even though insomnia patients should follow sleep hygiene recommendations, there is insufficient evidence to support the effectiveness of sleep hygiene alone for managing chronic primary insomnia [11]. For this reason, sleep hygiene should be combined with other interventions, such as biofeedback therapy (educating patients to control involuntary processes in their body such as muscle tension and blood pressure) [11].

Even though short-acting benzodiazepines or Z-drugs are recommended as the first-line pharmacological treatment for primary insomnia [11], the majority of participants rejected this recommendation to be included in the guidelines. A significant number of experts believed that benzodiazepines and Z-drugs should be used to treat primary insomnia only when it is severe, disabling, or causing extreme distress. A majority of the panellists also recommended prescribing these drugs in the first instance at the lowest effective dose. This dose should be used for maintenance and tapered off as determined by the prognosis [11]. Further, the specialists said that benzodiazepines or Z-drugs should not be prescribed for more than 4 weeks as recommended in the literature [23], and that switching from one hypnotic to another should be considered only if the patient experiences adverse effects from the drug they are using [22]. Importantly, most of the responders agreed that benzodiazepines should not be prescribed to known or suspected users of illicit drugs. According to the literature, exceptions may be made if the drugs are indicated as part of an opiate detoxification programme or prescribed under close monitoring and supervision by psychiatrists on an acute basis [22].

Overall, a consensus was reached for most of the recommendations with significant agreements between rounds. The e-Delphi technique was well received and external reviewers contributed extensive comments to support the development of guidelines. Although the authors believe that the contributions from the medical experts to the consensus statements are thoughtful and valuable, this study has some limitations. The absence of a face-to-face meeting might have deprived experts from exchanging important information, such as clarification of reasons for disagreements [24, 25].

## Conclusion

With little awareness about sleep disorders and the absence of evidence-based clinical guidelines on the management of insomnia, adults in the Saudi Arabia are at a higher risk of suffering serious consequences from the condition. It is imperative, therefore, that effective guidelines for treating insomnia and sleep disorders are developed in Saudi Arabia, particularly guidelines concerning the pharmacological management of primary insomnia with benzodiazepines and Z-drugs. Using the e-Delphi technique, this study developed evidence-based expert clinical opinions for using benzodiazepines and Z-drugs in managing insomnia, and such findings based on consensus statements, together with insomnia advocacy, reviewing service delivery across Saudi Arabia, and best-practice management of primary insomnia, can lead to optimal sleep disorder healthcare in Saudi Arabia.
